# Supplementary material for: Design and Psychometric Evaluation of the ‘Clinical Communication Self-Efficacy Toolkit’
Source: Int J Environ Res Public Health. 2019 Nov 16;16(22):4534. doi: 10.3390/ijerph16224534 (PMC6888055; doi:10.3390/ijerph16224534)
Supplement: Supplementary file 1 [file ijerph-16-04534-s001.zip › Supplementary Table S1.pdf]

Supplementary Table A1. Comments for improving readability and understandability

Participant or expert code	Tool	Item and Comment	Action taken
E2	PCC-SES	**Item 3:** *"I think it is important to reflect in the item that the healthcare professional should always clarify how much time he/she has before starting".*	The item was reworded and […] "before starting a conversation" was added at the end of the item.
E6	PCC-SES	**Item 8:** *"The authors should consider that some untrained people may not be conscious of the fact that they interrupt or influence patient's decisions".*	The item was reworded to include the expert's suggestion.
E10	PCC-SES	**Item 17:** *"I would include 'peculiarities' because sometimes is neither the other person's values, beliefs, opinions or concerns that we find difficult to accept, but the little things in the way they express, their tone, their manners…".*	The item was reworded to include the expert's recommendation.
E11	PCC-SES	**Item 4:** *"By saying 'Ask open questions' you give too much information away and nurses or nursing students may find this task too easy to achieve".*	The item was reworded and the term 'open' was substituted for 'right' as suggested by another expert.
E14	PCC-SES	**All items:** *"Please, include a full stop punctuation mark at the end of each item".*	A stop punctuation mark was added at the end of all items as recommended.
E17	PCC-SES	**Item 4:** *"Open questions should be changed for right questions to make people think".*	The item was reworded to include the expert's recommendation.
P19	PCC-SES	**Item 9 and 15:** *"I found item 9 and item 15 difficult to read".*	Item 9 was reworded and commas were added to item 15 to improve its readability.
P36	PCC-SES	**Item 13:** *"Item 13 is too long to read".*	No changes were applied to this item as it could not be split into two items and none of the information could be removed
P40	PCC-SES	**Item 9:** *"I do not understand what the word 'feedback' means".*	The term 'retroalimentación', which means 'feedback' in Spanish was added to the item.
E6	ISR-SES	**Item 24:** *"I recommend adding 'own' before 'self' to improve understandability".*	The item was reworded to include the expert's suggestion.
E14	ISR-SES	**All items:** *"Please, include a full stop punctuation mark at the end of each item".*	A stop punctuation mark was added at the end of all items as recommended.
P5	ISR-SES	**Item 28:** *"The 28th item is a bit too long and I had to read it various times to understand it".*	The item was modified and commas were added to improve its readability.
