# Supplementary material for: Design and Psychometric Evaluation of the ‘Clinical Communication Self-Efficacy Toolkit’
Source: Int J Environ Res Public Health. 2019 Nov 16;16(22):4534. doi: 10.3390/ijerph16224534 (PMC6888055; doi:10.3390/ijerph16224534)
Supplement: Supplementary file 1 [file ijerph-16-04534-s001.zip › Supplementary Table S2.pdf]

Supplementary Table S2

Spanish and English version of the 'Clinical Communication Self-Efficacy Toolkit'

CC-SET©

Name of the authors
José Manuel Hernández-Padilla
Alda Elena Cortés-Rodríguez
José Granero-Molina
Cayetano Fernández-Sola
Matías Correa-Casado
Isabel María Fernández-Medina
María del Mar López-Rodríguez

© **This toolkit is copyrighted.** Those individuals or organisations interested in using or reproducing the CC-SET (totally or partially) will have to seek permission from Dr. José Manuel Hernández-Padilla; email address: j.hernandez-padilla@ual.es

IMPORTANT NOTE
Please note that the English version of the CC-SET included in this appendix has not undergone a validation study. Therefore, its use in English speaking populations may require previous psychometric assessments.

TEST

Escala de autoeficacia en comunicación clínica (CC-SET)®

Hernández-Padilla JM, Cortés-Rodríguez AE, Granero-Molina J, Fernández-Sola C, Correa-Casado M, Fernández-Medina IM, López-Rodríguez MM.

Por favor, rellene los siguientes datos demográficos:

**Edad**		**Sexo**		**Curso**	
**Estudios completados antes de acceder al Grado de Enfermería**					
**¿Has recibido alguna formación formal en habilidades de comunicación?**				SÍ ☐	NO ☐
**¿Ha aprobado la asignatura de 'Ciencias psicosociales aplicadas a los cuidados de enfermería'?**				SÍ ☐	NO ☐
**¿Ha aprobado la asignatura de 'Enfermería de salud mental'?**				SÍ ☐	NO ☐
**¿Ha aprobado la asignatura 'Cuidados de enfermería en situaciones complejas de salud'?**				SÍ ☐	NO ☐

Este cuestionario ha sido diseñado para ayudarnos a entender mejor cuáles son las dificultades sentidas por los alumnos de enfermería a la hora de establecer un intercambio comunicativo eficaz.

Usando la escala dada, por favor indique su nivel de confianza en su capacidad para llevar a cabo las acciones que describen a más abajo, **sin cometer errores** y bajo las condiciones estresantes típicas de una situación clínica compleja (por ejemplo: paciente recién diagnosticado con una enfermedad terminal o un compañero que infravalora nuestro trabajo). Su respuesta será estrictamente confidencial y no será registrada con su nombre.

Utilice la escala dada y asigne un número que indique su nivel de confianza con las afirmaciones detalladas en el reverso de esta hoja.

0	10	20	30	40	50	60	70	80	90	100
Totalmente seguro que no puedo hacerlo					Moderadamente seguro que puedo hacerlo					Totalmente seguro de que puedo hacerlo

© **Estas herramientas están registradas.** Aquellas personas u organizaciones interesadas en usar o reproducir la CC-SET (total o parcialmente) deberán pedir permiso al siguiente autor: Dr. José Manuel Hernández-Padilla; email de contacto: j.hernandez-padilla@ual.es

UNIVERSIDAD DE ALMERÍA

Middlesex University

Escala de autoeficacia en comunicación centrada en la persona (PCC-SES)®

A la hora de establecer un intercambio comunicativo eficaz, estoy seguro de que siempre puedo…

		Confianza (0 -100)
**1.**	Crear un clima y/o entorno que facilite la efectividad del encuentro comunicativo	
**2.**	Presentarme y saludar a otros de manera apropiada, teniendo en cuenta la situación, el entorno y las peculiaridades de cada encuentro	
**3.**	Clarificar de manera adecuada el motivo del encuentro y cuál es mi disponibilidad para el mismo en cuanto a tiempo y otros recursos (por ejemplo, conocimiento o experiencia)	
**4.**	Formular correctamente las preguntas pertinentes para iniciar una conversación delicada dependiendo de cada situación	
**5.**	Adoptar y demostrar una actitud empática sin juzgar las preocupaciones de mi interlocutor	
**6.**	Reunir toda la información necesaria sobre las preocupaciones que la persona con la que establezco el contacto quiera compartir	
**7.**	Manejar de manera eficaz las pausas y los silencios durante cualquier encuentro interpersonal	
**8.**	Dar a la otra persona el tiempo y la oportunidad para expresarse sin influenciarla consciente o inconscientemente	
**9.**	Utilizar la técnica de feedback o retroalimentación en aras de garantizar la escucha activa y la clarificación	
**10.**	Animar y guiar a mi interlocutor para que identifique cuales son las redes de apoyo de las que dispone	

© Estas herramientas están registradas. Aquellas personas u organizaciones interesadas en usar o reproducir la CC-SET (total o parcialmente) deberán pedir permiso al siguiente autor: Dr. José Manuel Hernández-Padilla; email de contacto: j.hernandez-padilla@ual.es

**11.**	Facilitar y promover que mi interlocutor plantee una solución o establezca un plan para hacer frente a su situación	
**12.**	Ofrecerme como elemento de apoyo o ayuda sin dar consejos o resultar paternalista y clarificando mis límites	
**13.**	Resumir y clarificar los puntos clave del intercambio incluyendo los sentimientos y las preocupaciones del interlocutor; así como las posibles soluciones y el plan de acción acordados	
**14.**	Hacer un uso apropiado de los elementos no verbales de la comunicación durante cualquier tipo de interacción	
**15.**	Enviar mensajes completos a nivel de contenido, sentimiento y demanda, adaptándolos a las necesidades de mi interlocutor	
**16.**	Ser respetuoso con mi interlocutor independientemente de cual sea su actitud y sus preocupaciones	
**17.**	Aceptar, sin juzgar, las opiniones, valores, creencias, preocupaciones e individualidades de mi interlocutor	

Escala de autoeficacia en intercambio de información clínica y comunicación interprofesional (PIE-SES)®

A la hora de intercambiar información sobre un paciente de manera efectiva con un compañero, estoy seguro de que siempre puedo…

		Confianza (0 -100)
**18.**	Plantear claramente cuál es la situación por la cual se establece un contacto interprofesional acerca de un paciente concreto	
**19.**	Comunicar detalladamente los antecedentes del paciente por el cual se establece el contacto interprofesional	

© **Estas herramientas están registradas.** Aquellas personas u organizaciones interesadas en usar o reproducir la CC-SET (total o parcialmente) deberán pedir permiso al siguiente autor: Dr. José Manuel Hernández-Padilla; email de contacto: j.hernandez-padilla@ual.es

**20.**	Transmitir a otros profesionales la información recogida durante la valoración completa del paciente de manera organizada y siguiendo las recomendaciones internacionales	

**21.**	Proponer a otros profesionales recomendaciones congruentes y eficaces para abordar el problema del paciente	

**22.**	Defender asertiva y respetuosamente mi punto de vista ante las opiniones de otros profesionales	

**23.**	Escuchar y tener en cuenta las opiniones de otros profesionales para tratar de encontrar una solución conjunta al problema del paciente	

Escala de autoeficacia en comunicación intrapersonal y autorreflexión (ISR-SES)®

A la hora de comunicarme con mi propio self, estoy seguro de que siempre puedo…

Confianza (0 -100)

**24.**	Ser congruente y eliminar las diferencias entre mi self-real y mi self-ideal	

**25.**	Aceptar sin juzgar que el self de otros puede ser diferente al mío	

**26.**	Aceptar sin juzgar que otros pueden verme de manera diferente a como yo mismo me veo	

**27.**	Identificar y solucionar las diferencias entre cómo soy realmente y cómo me ven los demás	

**28.**	Hacer un uso apropiado de la autorrevelación como técnica para mejorar mis intercambios comunicativos, adaptándola a la persona, entorno y situación de manera adecuada	

© **Estas herramientas están registradas.** Aquellas personas u organizaciones interesadas en usar o reproducir la CC-SET (total o parcialmente) deberán pedir permiso al siguiente autor: Dr. José Manuel Hernández-Padilla; email de contacto: j.hernandez-padilla@ual.es

**29.**	Reaccionar de manera apropiada a las críticas de otros	

**30.**	Identificar y admitir posibles errores en mi forma de comunicarme	

**31.**	Reflexionar sobre mis interacciones comunicativas y hacer todo lo posible por mejorarlas en el futuro	

Por favor, use el siguiente recuadro para incluir cualquier comentario sobre el cuestionario.

Gracias por su colaboración.

© **Estas herramientas están registradas.** Aquellas personas u organizaciones interesadas en usar o reproducir la CC-SET (total o parcialmente) deberán pedir permiso al siguiente autor:

Dr. José Manuel Hernández-Padilla

Departmento de Enfermería, Fisioterapia y Medicina
Universidad de Almería
España
j.hernandez-padilla@ual.es

TEST

Clinical Communication Self-Efficacy Toolkit (CC-SET)®

Hernández-Padilla JM, Cortés-Rodríguez AE, Granero-Molina J, Fernández-Sola C, Correa-Casado M, Fernández-Medina IM, López-Rodríguez MM.

Please, complete the following:

**Age**		**Gender**		**Cohort** (Programme year)	
**Last academic qualification completed**					
**Have you ever attended any formal training on basic clinical communication skills?**				Yes ☐	No ☐
**When was the last time you attended a formal training on basic clinical communication skills?** (in months)					
**Have you ever attended a formal refresher on basic clinical communication skills?**				Yes ☐	No ☐
**When was the last time you attended a formal refresher on basic clinical communication skills?** (in months)					

This questionnaire is designed to help us gain a better understanding of the aspects of basic clinical communication that nursing students may find difficult to carry out in clinical practice.

Using the scale given, please rate how confident you are in doing the tasks described below, **without making mistakes and in the appropriate order**, under the potentially stressful conditions of the typically complex clinical environments. Your answer will be kept strictly confidential and will not be identified by name.

Rate your degree of confidence by recording a number from 0 to 100 using the scale given below.

0	10	20	30	40	50	60	70	80	90	100
Completely sure I cannot do at all					Moderately can do					Completely sure I can do

© **This toolkit is copyrighted.** Those individuals or organisations interested in using or reproducing the CC-SET (totally or partially) will have to seek permission from Dr. José Manuel Hernández-Padilla; email address: j.hernandez-padilla@ual.es

UNIVERSIDAD DE ALMERÍA

Middlesex University

Person-centred Communication Self-Efficacy Scale (PCC-SES)

In order to effectively communicate with other people, I am confident that I can always...

		Confidence (0 -100)
**1.**	Consciously choose the appropriate physical setting for my interactions before initiating them, taking into consideration the nature of each interaction.	
**2.**	Appropriately introduce myself and greet other people taking into consideration the particularities of each interaction.	
**3.**	Explain the reason why I am having an interaction with other people and clarify what my time availability is before starting a conversation.	
**4.**	Ask the right questions in order to initiate a difficult conversation, taking into consideration the particularities of each situation.	
**5.**	Demonstrate empathy without judging the other person's concerns.	
**6.**	Gather all the relevant information about the other person's concerns without forcing them to share it.	
**7.**	Make an appropriate and useful use of silence during my interactions with other people.	
**8.**	Give other people the opportunity to express themselves without consciously or unconsciously interrupting or influencing them.	
**9.**	Make an appropriate use of feedback in order to clarify what I have understood and to demonstrate that I am actively listening.	
**10.**	Encourage and guide people I am interacting with to identify what their support network may be without telling them myself.	

© **This toolkit is copyrighted.** Those individuals or organisations interested in using or reproducing the CC-SET (totally or partially) will have to seek permission from Dr. José Manuel Hernández-Padilla; email address: j.hernandez-padilla@ual.es

**11.**	Encourage other people to talk about what they think would help, giving them time to think about helpful solutions without suggesting my own ones.	

**12.**	Offer myself as a support element, asking the patient if I can help and clarifying my limits without being patronising or falling into giving advice that fits my needs and not the other person's needs.	

**13.**	Summarise and clarify the key points of the interaction, acknowledging the other person's feelings and concerns and including the suggested solutions and the agreed action plan.	

**14.**	Appropriately use all the non-verbal communication elements regardless of the situation.	

**15.**	Send messages that are always complete in terms of content, feelings and demand, adapting them to the needs of each person I interact with.	

**16.**	Be respectful with the other person regardless of their attitude and concerns.	

**17.**	Accept other people's opinions, values, beliefs, concerns and peculiarities without judging them.	

Patient clinical Information Exchange and interprofessional communication Self-Efficacy Scale (PIE-SES)

In order to effectively communicate with other healthcare professionals about a patient, I am confident that I can always…

		Confidence (0 -100)
**18.**	Clearly and briefly state the situation that led to establishing the contact regarding the patient I may need help with or advice for.	

**19.**	Communicate the patient background in a comprehensive, yet concise manner without forgetting any information.	

© This toolkit is copyrighted. Those individuals or organisations interested in using or reproducing the CC-SET (totally or partially) will have to seek permission from Dr. José Manuel Hernández-Padilla; email address: j.hernandez-padilla@ual.es

**20.**	Share with other healthcare professionals all the information collected during the patient assessment process in a structured, clear and concise manner.	

**21.**	Recommend appropriate and potentially effective solutions that may help with the patient's problem.	

**22.**	Justify and argue my point of view in an assertive and respectful manner when interacting with other healthcare professionals.	

**23.**	Listen to other professionals' opinions and take them into account in order to find a team solution to the patients' problem.	

Intrapersonal communication and Self-Reflection Self-Efficacy Scale (ISR-SES)

In order to effectively communicate with own self, I am confident that I can always…

		Confidence (0 -100)
**24.**	Be congruent with my own 'self' and eliminate the differences between my 'real-self' and my 'ideal-self'.	
**25.**	Accept without judging that other people's 'self' may be different to mine.	
**26.**	Accept without judging that other people may see me in a different way to how I see my own 'self'.	
**27.**	Identify and solve the differences between how I really am and how other people see me.	
**28.**	Appropriately use self-revelation as a technique to improve my interactions, adapting it to the other person, the environment and the situation.	

© **This toolkit is copyrighted.** Those individuals or organisations interested in using or reproducing the CC-SET (totally or partially) will have to seek permission from Dr. José Manuel Hernández-Padilla; email address: j.hernandez-padilla@ual.es

**29.**	Appropriately react to other people's criticism.	

**30.**	Identify and recognise potential mistakes in the way I communicate.	

**31.**	Reflect on my interactions with other people and do what I can to improve them in the future.	

Please, use the box below to add any comments you wish about this questionnaire.

Thanks for your cooperation.

© **This toolkit is copyrighted.** Those individuals or organisations interested in using or reproducing the CC-SET (totally or partially) will have to seek permission from the following author:

Dr. José Manuel Hernández-Padilla

Department of Nursing, Physiotherapy and Medicine
University of Almería
Spain
j.hernandez-padilla@ual.es
